# The Infirmary and Signs of Change

**Published:** 1913-04-05

**Authors:** 


### The Hospital

The Workers' Newspaper of Administrative Medicine and Institutional Life, Administration, National Insurance and Health.

No. 1396, Vol. LIV. SATURDAY, APRIL 5, 1913. PRICE ONE PENNY.

### THE INFIRMARY AND SIGNS OF CHANGE.

It is interesting to observe how both the voluntary hospital and the Poor-Law infirmary are sharing a common anxiety at the present time as to the future thrust upon them by recent legislation. For the problems opened up by the National Insurance Act are not solved for the voluntary hospital by a cautious admission of such cases only as "a competent panel practitioner" may be reasonably expected to meet. For the Poor-Law infirmary the Insurance Act is only the latest problem thrust upon it by a restive Legislature, and observant readers of our Special Number this week will not fail to note the anxiety, expressed from such diverse points of view, which is revealed by the expert writers on the chief infirmary questions of the hour. Nothing can illustrate this attitude more pointedly than the article on "Poor-Law Infirmary Publications," which gives, for instance, a place, and quite rightly, to a recent collection of sociological essays entitled "The Great State." The Poor-Law world knows itself to be on the brink of a great change, and as such is in a speculative mood, which turns at first in the face of so many diverse possibilities towards far-reaching generalisations. The two Reports of the Poor-Law Commission had this common ground between them, that extensive changes must come; and this confident verdict was promulgated before the problem of the nation's insured persons had forced certain lines of departure into the limelight.

The nature of the impending change can be summed up in the word "co-operation," and the divergencies arise only over which institutions shall co-operate, and which authorities shall be linked up. Hitherto the term "hospital" has always preferred to prefix an adjective, not merely to indicate the type of disease which it treats, but also the type of authority which controls it. The future, however, is tending to merge the latter distinctions into the public hospital, in which the voluntary hospital, while inevitably keeping its characteristic principle, will yet be brought into closer relation with the Poor-Law infirmary. At present the channel of intercommunication between the voluntary and the rate-supported institutions is unsatisfactory and troublesome from many points of view. The Hospital, however, as its catholic title implies, has always been conscious that the future must confirm and strengthen into a co-operative system the two types of institution.

The institutional world at large is awaiting the change, and the voluntary hospital, no less than the Poor-Law infirmary, is interested in the developments which await the latter. For as it is the cumulative effect of Parliamentary legislation which is making a co-operative change inevitable, the change will mirror itself in these institutions first which are under immediate State control. You cannot appropriate beds for insured consumptives from institutions under the Metropolitan Asylums Board, and condemn the phthisical wards of the infirmary as fitted primarily for advanced cases only, without finally bringing the dispensary, the chest hospital, the infirmary, and the sanatorium into co-operation. It is outside the scope of a leading article on this subject to indicate the lines which infirmary developments may probably take; that is done elsewhere under the suggestive title "From Infirmaries to Public Hospitals," where certain changes are dealt with in detail. Something more, however, is wanted than generalisation, and we have not hesitated to print a definite forecast of the infirmary's future, or to describe, as a specific example of co-operation, a "Practical Reform in the Casual Wards," in which the signs of change may be seen at work.

One of the difficulties of the Poor-Law infirmary medical superintendent is the isolation attaching to a post, the heavy duties of which are not counterbalanced by many opportunities for discussion and interchange of views with his colleagues in sister institutions; and this is all the more felt at a time when the uncertainty is so productive of discussion. We have, therefore, to thank those infirmary authorities who have co-operated with us to make this Special Number a summary of the tendencies of the hour; and we hope that their example may be followed by other infirmary officials. There is a great chance now for the focussing of infirmary opinion, which medical superintendents especially should take advantage of, since the small numbers of medical inspectors deprive them of an adequate channel for impressing their views on the Local Government Board, which will be the lever in the impending legislation. Then, again, there is the important question of construction, and both voluntary hospital and Poor-Law infirmary authorities will do well to consider Mr. Saxon Snell's comparison of the two types of construction which are required. It is interesting to have an architect's tribute to the guiding hand of the Local Government Board, to which the writer attributes the proportionately larger sites which he notes as characteristic of infirmary buildings. Further classification will, no doubt, affect the future designing of infirmaries, or public hospitals, as they may then be called, but it is instructive to note that while larger air-space per patient and smaller wards are to be found in voluntary hospitals, the demand for air-space has not been so recognised in relation to the site as a whole. The administrative block is a much larger building in a voluntary hospital than in an infirmary, and its encroachment on the site-space is often too great. On the brink of change it is well to take stock of the qualities which distinguish both institutions to-day.

